# Membrane Pathology in Schizophrenia: Implication for Arachidonic Acid Signaling

**DOI:** 10.1100/tsw.2002.870

**Published:** 2002-07-10

**Authors:** Jeffrey K. Yao, Ravinder K. Reddy

**Affiliations:** VA Pittsburgh Healthcare System, 7180 Highland Drive, Pittsburgh, PA 15206, USA; Department of Psychiatry, University of Pittsburgh Medical Center, USA; Department of Pharmaceutical Sciences, School of Pharmacy, University of Pittsburgh, Pittsburgh, PA 15213, USA

**Keywords:** schizophrenia, arachidonic acid, phospholipid metabolism, neurotransmission, phosphoinositide signaling system, eicosanoids, endocannabinoids, ethyl eicosapentaenoate supplementation

## Abstract

Schizophrenia is a major mental disorder with no clearly identified pathophysiology. A variety of theories has been proposed to explain the pathophysiology of schizophrenia. One approach that is finding empirical support is the investigation of membrane composition and function. Evidence to date suggests that there are defects in phospholipid metabolism and cell signaling in schizophrenia. Specifically, low levels of arachidonic acid (AA)–enriched phospholipids have been observed in both central and peripheral tissues. It is well known that changes in membrane composition are associated with a variety of functional consequences. Since AA has many key roles in neural functioning, understanding its significance for the pathophysiology of schizophrenia may lead to novel approaches to improving treatment of schizophrenia. The purpose of this review is thus to explore some of the roles of AA signaling in biological, physiological, and clinical phenomena observed in schizophrenia.

## INTRODUCTION

Schizophrenia is a complex disorder. Research over the last century has suggested that neuronal maldevelopment, impaired neurotransmission, intrauterine viral infections, autoimmune dysfunction, and many other mechanisms may underlie the pathophysiology of schizophrenia. There are a large variety of seemingly disparate biological findings[1], possibly due to etiologic heterogeneity. This would suggest that there are one or more (but very few) common pathogenetic pathways that lead to the syndromes of schizophrenia. Thus, there is need to identify pathological process(es) that can explain many of the clinical and biological features in schizophrenia.

There is substantial evidence for both peripheral and central membrane abnormalities in patients with *chronic* schizophrenia, and a relative paucity of such evidence in early schizophrenia[2]. One of the key findings in the RBC membrane is decreased polyunsaturated fatty acids (PUFAs), particularly arachidonic acid (AA). Further, alterations in AA are associated with membrane dysfunction of clinical relevance. This defect appears to be independent of neuroleptic treatment (based on findings from drug free and neuroleptic-naive patients), and is associated with illness severity. The primary mechanisms that may lead to reduced membrane AA are increased phospholipid hydrolysis and/or decreased incorporation. The accelerated breakdown of membrane phospholipids is indirectly supported by the $^{31}$P Magnetic Resonance Spectroscopy ($^{31}$P MRS) findings as well as direct measurements of phospholipid fatty acids in the brains of schizophrenic patients. Supplementation with essential fatty acids (EFA) is associated with increased membrane PUFAs and with improved clinical state.

## MEMBRANE DEFECTS IN SCHIZOPHRENIA

### Decreased Membrane Phospholipids

Early studies indicated that there were a variety of alterations in levels of phosphatidylcholine (PC), phosphatidylserine, and phosphatidylinositol (PI), and consistent decreases of phosphatidylethanolamine (PE) in RBC membranes from patients with psychoses[3]. Phospholipid abnormalities have also been found in medication-free schizophrenic patients[4], and decreases in all four key membrane phospholipids were found in fibroblasts from neuroleptic-naive schizophrenic patients[5].

$^{31}$P MRS has been shown to reveal important insights into the metabolism of cell membranes. Phosphomonoesters (PMEs) are the precursors, and phosphodiesters (PDEs) the breakdown products of membrane phospholipids. PME and PDE resonances reflect membrane turnover and may differ between healthy and pathological states. Phospholipids themselves constitute a large part of the broad resonance underlying the PDE and PME peaks. Pettegrew et al.[6,7] have shown significant reduction of PMEs and significantly increased levels of PDEs in the frontal cortex of neuroleptic-naive first-episode schizophrenic patients. They have proposed that changes in membrane phospholipids may be related to molecular changes that precede the onset of clinical symptoms and brain structural changes in schizophrenia[7]. Other investigators have also reported similar findings in membrane phospholipid perturbations in both acutely and chronically ill patients[8,9,10,11,12]. Direct evidence of decreased phospholipid levels comes from postmortem study of the caudate[13] of schizophrenic patients relative to normal controls, findings that may underlie an increased phospholipid breakdown observed using $^{31}$P MRS.

### Decreased PUFAs

Significant reductions in plasma AA (20:4 n-6) and linoleic acid (18:2 n-6), a precursor of AA, but an increase of total n-3 fatty acids in schizophrenic patients from three geographic regions has been shown[14]. Other investigators have reported decreases in RBC membrane PUFAs in schizophrenia[15,16,17,18]. Moreover, decreases of RBC-PUFAs were not affected by haloperidol treatment[17]. Reduction of AA in skin fibroblasts has been found in first-episode schizophrenic patients[19].

Recently, a robust reduction of total PUFAs was found in schizophrenic brains, relative to control brains[13]. This is consistent with the observed reduction of membrane PE and PC. Specifically, the decrease of PUFAs was largely attributable to reductions in AA, 20:4(n-6) and, to a lesser extent its precursor, 18:2(n-6) and 20:2(n-6). A similar decrease of 20:4(n-6) was also found in the frontal cortex of schizophrenic patients[20]. These data are in accordance with findings in plasma and RBC membrane fatty acids. In light of findings of membrane defects in a variety of peripheral cell types (platelets, RBC, and fibroblasts), it has been proposed that in schizophrenia these membrane defects may be generalized in the body, thus being detectable in both extraneural tissues as well as the brain[21].

## AA SIGNALING

### Phospholipid Hydrolysis and Neurotransmission

In the brain, AA and its metabolites are considered as the intracellular second messengers. It is known that many neurotransmitters can potentiate AA release through a receptor-dependent hydrolysis of membrane phospholipids, which suggests that the receptor-mediated AA release may participate in neuronal signal transduction. Therefore, the depleted AA resulting from an increased phospholipid breakdown has been considered as a common factor that regulates prostaglandin biosynthesis, neurotransmission, and neuronal deficits in schizophrenia[22].

### *Increased Phospholipids Degradation*

Phospholipase $A_2$ ($PLA_2$) is a key enzyme responsible for the breakdown of membrane phospholipids. It is enriched in neuronal membranes. Increased cytoplasmic $PLA_2$ activity has been found in serum of drug-free schizophrenic patients[23,24,25]. Such increases in serum $PLA_2$ activity, however, were also found in patients with other psychiatric disorders[25], questioning the specificity of this finding to schizophrenia. Albers et al.[26] found no significant differences of serum $PLA_2$ activity between neuroleptic-naive schizophrenics and normal controls. These discrepancies may be due to the differences in assay procedure and the heterogeneous class of extracellular $PLA_2$ [27]. Gattaz et al.[28] showed that the intracellular membrane-bound platelet $PLA_2$ activity was significantly higher in schizophrenic patients than in normal and psychiatric controls, with no significant differences between normal and psychiatric controls. It is thus unlikely that the increased platelet $PLA_2$ activity in schizophrenia results from nonspecific stressors. Furthermore, haloperidol treatment reduced platelet $PLA_2$ activity to control levels. Other neuroleptics also inhibit $PLA_2$ activity[29,30,31].

Moreover, a significant increase in spontaneous contralateral circling is seen following injection of bovine $PLA_2$ intranigrally into rats[32], which is alleviated by neuroleptics. Similar results were also obtained by Cadet and Lohr[33], who further demonstrated that intracerebral injection of $PLA_2$ can reduce dopaminergic activity. $PLA_2$ is known to inhibit dopamine-sensitive adenylate cyclase activation[34] and to reduce the [$^3$H]spiperone binding to dopamine receptors[35].

### *Neurotransmission*

#### *Decreased Dopamine Transport*

Changes in membrane dynamics can affect transmembrane processes[36]. The function of the DA transporter receptor (DATR) is highly influenced by the lipid composition of membrane environment. Decreased DATR density has been found in cortical areas with high metabolic activity in schizophrenic brains[37]. Further, both n-6 and n-3 series of PUFAs may be involved in the presynaptic receptor control of dopamine release[38]. Dopamine $D_2$ receptors have been shown to act synergistically with $Ca^{2+}$ stimuli to release AA from membrane phospholipids[39]. Further, dopamine $D_2$ receptors potentiate AA release via activation of cytosolic, AA-specific $PLA_2$[40]. Thus, decreased AA can alter dopaminergic transmission.

### *Serotonin (5-$HT_2$) Dysfunction*

There is abundant evidence that 5-$HT_2$ receptors in the brain play a regulatory role in behavior[41]. 5-HT stimulates the release of AA in hippocampal neurons through the activation of $PLA_2$ that is independent of inositolphospholipid hydrolysis[42]. Thus, 5-HT may potentially mediate some pathophysiological processes through receptor-stimulated AA or eicosanoids. We have demonstrated that drug-free schizophrenic patients exhibit reduced physiologic responsivity mediated through the platelet 5-$HT_2$ receptor complex, which can be modified by haloperidol treatment[43].

### *Impaired Glutamatergic Neurotransmission*

Activation of N-methyl-D-aspartate (NMDA) by glutamate stimulates $PLA_2$ activity to release AA, and subsequently facilitates long-term potentiation of glutamate synapses in the hippocampus, probably by a prolonged inhibition of glutamate uptake into glial cells[44]. Decreased availability of AA may lead to an impaired glutamatergic neurotransmission. A dopamine-glutamate imbalance has been postulated as one pathogenic mechanism of schizophrenia[45]. Neuroleptic drugs that block dopamine receptors may also enhance the glutamatergic neurotransmission.

## Hyperactivity of PI Pathways

Early studies indicated that the receptor-stimulated hydrolysis of inositol phospholipids, particularly phosphatidylinositol 4,5-bisphosphate [PI-4,5-$P_2$], is initiated by a specific phospholipase C (PLC)[46,47]. A specific GTP-binding protein appears to be responsible for transducing the activated receptor through plasma membrane and activation of PLC. The resulting diacylglycerol (DAG) and inositol 1,4,5-triphosphate [1,4,5-$IP_3$] lead to activation of protein kinase C (PKC) and elevation of cytosolic $Ca^{2+}$, which provide molecular links between extracellular signals and intracellular events[47,48]. Thus, both DAG and $IP_3$ are second messengers that generally act in concert.

Quantitative determination of inositol phosphates provides direct evidence for PI hydrolysis by PLC in intact cells[49]. Increased turnover of platelet PI was found in both drug-treated and drug-free patients[50,51,52] but not drug-naive patients[51]. The increased production of $IP_3$ may be due to an increase in the precursor, PI-4,5-$P_2$, associated with a desensitization of the intracellular $IP_3$ receptor by neuroleptics[50]. On the other hand, Zilberman-Kaufman et al.[53] have reported an increased inositol-1-phosphatase in RBC of chronic schizophrenic patients. They interpreted that the increased enzyme activity might compensate physiologically for a deficiency of inositol in these patients. A trial of inositol therapy[54], however, showed no measurable psychoactive effect in chronic schizophrenic patients treated with neuroleptics.

In human platelets, DAG can be produced within 5 s of thrombin activation[55]. The newly formed DAG is either phosphorylated to phosphatidic acid (PA) by a specific DAG kinase[56] or cleaved to monoacylglycerol (MAG) by DAG lipase (see below). Both reactions may be considered as a termination for DAG intracellular signaling[57]. We have reported increased formation of DAG in thrombin-stimulated platelets of both haloperidol-treated and drug-free patients[58], consistent with the findings of Kaiya et al.[59]. Using [$^{32}$P]orthophosphate as a precursor, we have previously demonstrated that thrombin-induced formation of platelet PA was substantially higher in schizophrenic patients than in normal controls[52]. Therefore, the increase in thrombin-induced platelet DAG may be due, at least in part, to an increased PI turnover in schizophrenic patients[51,52]. Although schizophrenic patients with abnormal PI turnover appeared to have a better outcome than other patients[59], whether the second messenger (DAG) accumulation is correlated with clinical response in schizophrenia requires further investigation.

The functionality of the PI intracellular signaling has further been investigated in the postmortem human brain[60,61,62,63]. Using GTPγS to assess the activation of the G protein coupled with the stimulation of PLC, Jope et al.[64] have further demonstrated selective increases in PI signaling activity and Gαo levels in postmortem brain from schizophrenic subjects. This finding is in partial agreement with the result of Wallace and Claro[63] showing a similar but not statistically significant increase in GTPγS-stimulated PI hydrolysis in prefrontal cortex of schizophrenic patients compared with controls. Thus, taken together with the findings in platelets, there is ample evidence of hyperactivity of the PI signaling system in schizophrenia that is markedly different from diseases with major depression and bipolar mood disorder showing a decreased activity of G protein–mediated PI hydrolysis[62,65].

## Eicosanoids

In addition to the formation of second messengers, the newly released AA from membrane phospholipids can be converted to a variety of biologically active metabolites, which are collectively referred to as eicosanoids, through the concerted reactions of cyclooxygenase (COX) and lipoxygenases. Eicosanoids are the potent messengers, which modulate neural cell function as well as involve in pathophysiological processes[66]. Since AA is the major C20 PUFAs in mammalian tissues, the prostaglandin-2 ($PG_2$) and thromboxanes-2 ($TX_2$) series are the predominant classes of eicosanoids. Studies involving the inhibition of COX by nonsteroidal anti-inflammatory drugs have revealed the significance of $PG_2$ in the regulation of nerve conduction, neurotransmitter release, inflammation, pain, fever, immune responses, and apoptosis.

In schizophrenia, there are reduced levels of AA in membrane phospholipids that could conceivably lead to a decreased synthesis of eicosanoids. A deficiency of prostaglandins has previously been related to schizophrenia[67]. One of the AA metabolites, $PGD_2$, mediates vasodilatation during the inflammatory response. Therefore, the reduced AA availability may in part to explain a variety of clinical observations in schizophrenia that are usually ignored by the receptor-based etiological hypotheses[68]. For example, in schizophrenia, there appears to be a lower risk of arthritis and other inflammatory diseases[69], greater resistance to pain[70,71], and remission of psychosis during fever has been observed[67]. These effects could be secondary to a reduced eicosanoid signaling.

## Endocannabinoid System

### *Cannabinoid and Schizophrenia*

Δ9-Tetrahydrocannabinol (Δ9-THC), the psychoactive ingredient from *Cannabis saliva* or marijuana[72], has been known for centuries to cause acute euphoria, altered time perception, dissociation of ideas, paranoia, motor impairment, and occasional hallucinations[73]. The behavioral effects of cannabinoids vary in humans and are mainly dose dependent. With severe intoxication, a variety of cognitive and behavioral functions including memory, attention, reaction time, concept formation, motor coordination, and perception, can also be affected. Thus, a possible relationship between THC use and the development of psychosis has been explored in the early 1970s[74,75]. In fact, many clinical symptoms from the cannabis users resemble negative symptoms in patients with acute schizophrenia[76]. Later, Chopra and Smith[77] also reported psychotic episodes following cannabis use in a group of East Indian marijuana users. Particularly, those with "schizoid" personality features exhibited full-blown schizophrenic symptoms during the period of intoxication. Subsequently, Thacore and Shukla[78] have demonstrated an association of the paranoid psychosis with long-term cannabis use in 25 patients with paranoid schizophrenia. Chaudry et al.[79] and Abood and Martin[73] have further shown that moderate to severe THC intoxication closely mimics many of the positive and negative symptoms of schizophrenia. However, whether cannabis psychosis is a distinct clinical entity remains unclear[80].

Although the role of cannabis in the development of schizophrenia is unclear, the drug may modify the course of an already established illness[80]. For example, cannabis can affect the severity of schizophrenic symptoms[81], increase relapse rates[82], and decrease the efficacy of antipsychotic drugs[83]. In a longitudinal study of 45,570 subjects with 15 years follow-up, Andreasson et al.[84,85] have demonstrated that individuals with cannabis consumption have increased risk up to sixfold of developing schizophrenia as compared to normals or other drug users.

On the other hand, there were no differences in psychotic symptoms between schizophrenic patients with and without cannabis abuse. However, decreased negative symptoms were observed in cannabis users[86,87].

### *Tetrahydrocannabinol (THC) Ligands*

In 1988, Devane et al.[88] first characterized cannabinoid receptor in rat brain membranes. Recently, two endogenous THC ligands, anandamide[89] and 2-arachidonoylglycerol (2-AG)[90,91], have been discovered in the brain. Both anandamide and 2-AG are derivatives of AA. Anandamide is synthesized by the "transacylase-phosphodiesterase pathway"[92], which transfers the sn-1 linked acyl group of a glycerophospholipid to the ethanolamine group of PE. On the other hand, 2-AG is formed through the PLC-mediated degradation of PI as well as other membrane phospholipids. The resulting DAGs are hydrolyzed by sn-1-DAG lipase to produce 2-MAG, including 2-AG.

Because AA is primarily esterified at the sn-2 position of glycerophospholipids, 2-AG is often a major component of cellular MAG. On the other hand, NAEs that derive their fatty acids from the sn-1 position contain only trace amounts of anandamide (20:4 NAE) in virtually all mammalian cells and tissues, as well as in plasma. In brain, however, 2-AG is present in amounts 170 times greater than anandamide[91].

Given the localization of endogenous cannabinoid receptor ($CB_1$) system in brain areas (i.e., cortical and limbic structures) known to be implicated in schizophrenic brain pathology[93,94], it is plausible that dysfunction of $CB_1$ system with endogenous ligands be associated with the pathophysiology of schizophrenia. Moreover, there is a close interaction between $CB_1$ and dopaminergic systems. Cannabinoid agonists such as THC and the endogenous ligands, anandamide and 2-AG, can modulate the dopaminergic system[95,96,97]. Our lab[2] as well as others[16,18,20] have reported abnormalities in membrane AA from patients with schizophrenia. Since AA is the precursor of anandamide and 2-AG, it is possible that there exists a dysfunction of $CB_1$ system in schizophrenia.

Recently, Berdyshev et al.[98] have shown that production of 2-AG is markedly elevated during platelet activation. Taken together from the above observations, it is likely that an increased 2-AG resulting from the hyperactivity of PI signaling system and DAG second messenger formation enhance the activation of cannabinoid receptor system in schizophrenia. This hypothesis is in agreement with the view[99] that the ability of platelets to generate 2-AG and release it into the circulation may affect cannabinoid receptors in the brain.

## CLINICAL RELEVANCE

### Clinical Correlates

Low levels of RBC AA have been associated with prominent negative symptoms[16] and persistent positive symptoms[100]. In drug-free chronic schizophrenic patients, we found linoleic acid levels were inversely correlated with psychosis severity, suggesting the possibility of a defect in the conversion of linoleic acid to AA. Although these studies were conducted in chronic schizophrenic patients, no effects of long-term typical neuroleptics on AA levels were observed. Reduced incorporation of AA has been found in schizophreniform and untreated schizophrenic patients[101]. Recently, we have also demonstrated a decreased level of PUFAs in RBC membranes of first-episode neuroleptic-naïve schizophrenic patients[102], suggesting membrane pathology is present also early in the course of illness. Thus, a specific membrane defect may be associated with poor outcome (prominent negative and persistent positive symptoms), and may be present early in illness.

## Tardive Dyskinesia (TD)

Approximately 20% of patients receiving neuroleptics long term develop TD, often in those patients who exhibit the deficit syndrome. Vaddadi et al.[15] found that psychiatric patients (primarily schizophrenics) with TD had significantly lower RBC PUFA of both n-6 and n-3 series. The reduction of PUFA progressed as the severity of the TD worsened. These PUFA abnormalities were thought to be due to dietary factors, drug therapy, and hospitalization status[103]. Low plasma AA levels may also increase the risk for dyskinesia in the general elderly population. Nilsson et al.[104] found in 446 older Swedish men that the rate of dyskinesia was 15.1%, with a robust association with low AA. Zubenko and Cohen[105] have shown that platelet membrane fluidity, a direct measure of membrane function, is altered in patients with TD.

## Niacin-Induced Flushing

Facial flushing is induced by 200 mg of oral niacin in about an hour in the majority of normal and depressed subjects[106]. Schizophrenic patients with low levels of AA fail to flush in response to oral niacin[107]. Some patients with schizophrenia, particularly those with the negative or deficit syndrome, fail to flush facially in response to niacin[106,108,109]. Niacin flushing is dependent on the release of prostaglandin $E_1$ from 18:3(n-6)[108] and of prostaglandin $D_2$ from AA[110]. It is known that $PGD_2$ is involved in the vasodilation during the inflammatory response. Since $PGD_2$ is synthesized from the AA released from membrane phospholipids, the reduced niacin-induced flushing may be due to reduced membrane phospholipid AA. It is conceivable that endocannabinoid overproduction may downregulate prostaglandin biosynthesis, since both pathways share the same precursor, AA.

## Later Onset of Illness in Female Patients

Males are more likely to develop schizophrenia at an earlier age, whereas females tend to have the onset of illness[111,112,113]. Females may have a lower requirement than males for EFA, possibly because of the presence of estrogen[114,115], and thus might be expected to retain membrane AA in early life better than males. Such an advantage, however, would disappear after menopause[21].

## Other Mental Disorders

Membrane phospholipid abnormalities have been observed in dyslexia and attention deficit hyperactivity disorder (ADHD)[116,117]. Clinical features common to schizophrenia and these developmental disorders include language system and attention deficits[118,119]. Thus, PUFAs membrane deficits may explain many biological, physiological, and clinical consequences observed in schizophrenia[68].

## THERAPEUTIC EFFICACY (AA, AN INDEX OF THERAPEUTIC EFFECT?)

The mainstay of treatment of schizophrenia is an antipsychotic agent, primarily the typical and atypical neuroleptics. However, the response to current treatments is variable and far from acceptable, with only 60% of schizophrenic patients responding favorably. Treatment nonresponders frequently have prominent cognitive deficits and negative symptoms. Thus, any feasible method that can mitigate the persistent positive and negative symptoms, as well as the cognitive deficits can improve outcome.

### PUFA Supplementation: Correlation between Clinical State and RBC AA

Investigating EFA metabolism has proved fruitful for generating and testing novel etiologic hypotheses and new therapeutic agents for schizophrenia[120]. The early studies of PUFA supplementation were aimed at TD without significant success[121,122,123,124].

However, dietary supplementation with eicosapentaenoic acid (EPA), a precursor of docosahexanoic acid (DHA), has shown promising results in decreasing some of the clinical symptoms of schizophrenia[18,125,126,127], as well as cognitive impairments associated with dyslexia and ADHD[116,117]. There were no treatment-related side effects or adverse biochemical or hematological effects[128]. In a multicenter study with EPA treatment, Peet and Horrobin[128] have shown that patients on 2 g/day not only showed significant reduction of plasma triglycerides levels which had been elevated by clozapine, but also clinically improved. In addition, there was a positive correlation between clinical improvement and rise in RBC AA concentration.

### Atypical Antipsychotic Treatment: Ability of Apolipoprotein D to Bind AA

Clozapine, a widely used atypical antipsychotic drug, has been shown to be effective and relatively well tolerated in acute and long-term treatment of patients with schizophrenia, especially those who have not responded to conventional pharmacotherapies[129]. In contrast to typical neuroleptics, the dopamine $D_2$ receptor antagonists, clozapine exhibits an expanded spectrum of affinity for other neurotransmitter receptors, including 5-$HT_2$, histamine, muscarinic and adrenergic receptors[130]. However, the improved clinical efficacy of clozapine may not be attributed exclusively to its drug-blocking profile.

In order to understand the molecular mechanisms of clozapine's potentially unique actions, a PCR-based differential gene expression method, TOGA (Total Gene Expression Analysis) was used to identify genes whose expression may be altered by clozapine administration[131]. Following screening procedures, a regulation of apolipoprotein D (apoD) has been unveiled in the mouse brain resulting from clozapine treatment. Increases in apoD expression were detected in white matter regions, including corpus callosum, internal capsule and optic tract, and gray matter regions, including the striatum and globus pallidus[131]. These results implicate apoD in the mechanisms of action of clozapine.

*In vitro*, apoD has been shown to bind and transport ligands including AA, cholesterol, a human axillary odorant, heme-related molecules and steroid hormones, such as progesterone and pregnenolone[132,133,134,135,136,137]. Despite the ability of apoD to bind these small molecules, the physiological ligand(s) has yet to be identified definitively and it is possible that apoD has multiple, tissue-specific physiological ligands that may function differently under normal and pathological circumstances. Interestingly, the ability of apoD to bind AA implicates it in pathways associated with membrane phospholipid signal transduction and metabolism. Clozapine has been shown to increase AA and DHA levels in RBC phospholipids from schizophrenic patients[107,138]. It is likely that the elevated apoD levels such as those caused by clozapine may be linked with the observed clozapine-induced increase in AA, and possibly be beneficial for patients.

### Mood Stabilizers: Inhibition of AA-Specific Cytosolic $PLA_2$ by Lithium or Valproic Acid

Although lithium and valproic acid have been shown effectively in treating both phases of bipolar disorders, it is not clear whether a common biochemical pathway underlie their action. Because of lithium's ability to inhibit inositol-1-phosphatase, the sensitivity of receptor systems that utilize PI may be altered by prolonged exposure to lithium[46,139]. Thus, the therapeutic actions of lithium have been targeted toward those neuronal systems in which the receptor-mediated PI turnover is most active[140,141,142]. Changes in the second messengers may result in prolonged effector stimulation that could underlie mania. Similarly, excessive receptor desensitization may lead to depression. However, the chronic lithium treatment at relevant clinical doses only exhibit modest or no changes in PI turnover[143]. In addition, other membrane phospholipids were also altered in rat brain following chronic lithium treatment[144].

Following intravenous infusion of radiolabeled palmitic acid and AA in awake rats treated chronically with lithium, Chang et al.[145] have demonstrated decreased turnover rate of AA second messenger in brain phospholipids by up to 80%. In contrast, lithium had a minimal effect on turnover of palmitic acid. Decreased AA turnover was associated with a down-regulation of gene expression and protein levels of AA–specific cytosolic phospholipase $A_2$ ($cPLA_2$), but not with the intracellular $PLA_2$[146]. Similarly, valproic acid had the same effect on AA turnover, although it did not alter the $cPLA_2$ protein levels[147]. Taken together, both lithium and valproic acid have the same therapeutic action in reducing brain turnover of AA, probably via a different mechanism.

## CONCLUSION

There is accumulating evidence of membrane fatty acid compositional deficits in schizophrenia. These deficits have been found in peripheral tissues and the brain, in both neuroleptic-naïve and treated patients. The membrane fatty acid deficits are specific, primarily reductions in the n-3 and n-6 classes. Although much of the attention by early investigators has on n-3 fatty acids, increasing attention is being paid to the potentially important role that AA may play in the pathophysiology of schizophrenia. Recent clinical trials suggest that AA may be an index of therapeutic, and possibly pharmacological, action. AA has a key role in many physiological mechanisms that mediate signal transduction. Thus, future studies should clarify the specific role that AA has in the development, illness presentation and treatment of schizophrenia and related disorders.

## ACKNOWLEDGEMENTS

This study was supported in part by the Office of Research and Development (Merit Review, JKY), Department of Veterans Affairs, the Highland Drive VA Pittsburgh Healthcare System, and research grants from the National Institute of Mental Health (MH43742 and MH58141).
